# Validation of a gender-specific binary depression screening version (GIDS-15) in two German samples

**DOI:** 10.3389/fpsyt.2025.1469436

**Published:** 2025-04-24

**Authors:** Jan S. Pellowski, David D. Ebert, Hanna Christiansen

**Affiliations:** ^1^ Institute for Sex Research, Sexual Medicine and Forensic Psychiatry, University Medical Center Hamburg-Eppendorf, Hamburg, Germany; ^2^ Department of Psychology, Philipps University Marburg, Marburg, Germany; ^3^ School of Medicine and Health , Department Sport and Health Sciences, Technical University Munich, Munich, Germany; ^4^ Department of Clinical Child and Adolescent Psychology, Philipps University Marburg, Marburg, Germany; ^5^ Kinder- und Jugendlichen-Psychotherapie-Ambulanz Marburg (KJ-PAM)/Child and Adolescent Outpatient Clinic Marburg, Marburg, Germany; ^6^ Deutsches Zentrum für Psychische Gesundheit (DZPG)/German Center for Mental Health, Marburg, Germany

**Keywords:** depression, male depression, gender inclusive depression scale, screening version, binary gender-specific diagnostics

## Abstract

**Objective:**

Findings from depression research increasingly suggest a gender-dependent clinical appearance of relevant symptoms. At the same time, there is a lack of gender-sensitive screening procedures in clinical practice to better identify hidden depression in men. The present study examines the factor structure and psychometric characteristics of the translated version of the Gender Inclusive Depression Scale (GIDS) based on two large German-speaking mixed-sex samples, and assess sex and age effects.

**Methods:**

The preliminary exploratory validation of the German GIDS version was initially carried out using exploratory factor analysis with an online recruited non-clinical sample (*N* = 1173). The established factor structure was replicated with confirmatory factor analysis in a separate sample (participants of an alcohol prevention study; *N* = 418). Psychometric properties were calculated.

**Results:**

The exploratory factor analysis resulted in a 5-factor solution, and was confirmed in the second analysis. A screening version comprises 15 items. Overall, psychometric properties are satisfactory, with only two subscales (aggressiveness, substance use) with critical values. The majority of sex effects could be established.

**Conclusions:**

The GIDS-15 is a solid, multidimensional depression screening instrument that should be complemented by a gender assessment tool. There is further evidence that the inclusion of additional criteria alters the gender ratio in depression screening. After further studies to validate the GIDS-15, implementation in primary care could be indicated. Gender beyond the binary should be analysed in further studies.

## 1 Introduction

It is a consistently replicated finding in epidemiological health research that the lifetime prevalence rates of depression are at least twice as high in females compared to males (1–3). Given that suicide rates are three times higher in men than in women, and since suicidality is associated with depression (4, 5), such sex differences have been repeatedly questioned. In the ensuing scientific discourse, the influence of gender role stereotypes – independent of biological sex – in shaping the perception and of mental health disorders entities has been criticially examined (6–10). In this context, gender - in contrast to biological sex - is understood as the psychosocial gender associated with ideas about one’s own gender identity as well as gender role norms, attitudes and behaviours. Accordingly, a possible underdiagnosis and undertreatment of depression in men could result from an orientation towards socially determined gender stereotypes and gender-specific coping strategies (11–13). This assumption is supported by the results of a suicide prevention programme of the International Committee for Prevention and Treatment of Depression. This programme was carried out in the early 1980s on the Swedish island of Gotland to qualify practising doctors in the diagnosis and treatment of depressive disorders (14). The fact that suicide rates were significantly reduced in women, but remained almost unchanged in men, was the impetus for the development of the concept of male depression (15, 16). It states that depression occurs with similar frequency in both genders, but that men show different symptoms (i.e. mostly externalising) than women, based on their socially determined gender role. However, male-typical symptoms are not yet included in the standard diagnostic criteria of the leading diagnostic classification systems, the International Statistical Classification of Disease and Related Health Problems (17), the Diagnostic and Statistical Manual of Mental Disorders (18), or in frequently used questionnaires (19, 20). Instead, these classic classification systems and diagnostic tools emphasise a persistently depressed mood, a loss of interest and a lack of pleasure as the main criteria and changes on an affective, cognitive and somatic-visceral level as secondary criteria. Essentially, typical female symptom descriptions are recorded. However, depression is a heterogeneous disorder. Field trials studies on rater agreement for major depression according to DSM-5 revealed questionable reliability between raters (21). Apparently, symptoms cannot be easily differentiated even by experts. In terms of artefact theory, a gender bias in the diagnosis of depression is therefore assumed (22, 23), which could mask depression in men and enable misdiagnosis.

An increasing number of studies in recent years support this assumption and show that men with depression express different symptoms than women (24). For example, symptoms such as irritability, aggressiveness, substance abuse or risk-taking behaviour are mentioned (24–28). In line with the male gender role stereotype, these markers appear to be accepted behaviours for resolving conflicts and dealing with male distress (29–31). Therefore, externalising symptoms of depression (such as aggressiveness, risk-taking behaviour or substance abuse) have become increasingly important as potential indicators of depression in men who follow masculinity norms (32). At the same time, findings indicate that externalising depressive symptoms are also found in women (25, 33). It is assumed that this finding reflects the fact that there is an increasing social acceptance of aggression in women (33). It is argued that male norms are also becoming increasingly relevant for women and could therefore also be responsible for male-typical symptoms of depression in women. To take this into account, the term “masculine depression” has recently been proposed as a depression subtype instead of male depression (10). The importance of considering gender stereotypes in the ratings of certain symptoms is therefore crucial in this area (29, 34, 35). In order to translate research findings into clinical practice, gender-sensitive measurement tools for depression are increasingly being developed (36). For example, the Gender Inclusive Depression Scale (25) was used for the first time in an internationally recognised study (25). The GIDS takes alternative, male-typical symptoms of depression (such as aggressiveness, substance use, risk behaviour, stress experience) into account while also incorporating the conventional DSM-IV diagnostic criteria for major depression. In the study, sex differences in the prevalence of the sample disappeared when using the GIDS, with men reporting more risk behaviour, alcohol consumption, anger attacks and hyperactivity than women (25). Other gender-specific depression instruments have also been developed in the past. The first and probably most frequently examined inventory for the assessment of depression in men is the Gotland Male Depression Scale (GMDS) (15, 16). It includes questions among other things about stress, anxiety, aggressiveness, alcohol and drug abuse and hyperactivity, as well as conventional symptoms of depression. The Masculine Depression Scale (MDS) (37) includes for example stress, anxiety, feeling overburdened, aches and pains, or need for autonomy in addition to the classic depression symptoms. (26) also developed the Male Depression Risk Scale (MDRS-22) based on the assumption that men who identify strongly with masculinity norms also exhibit externalising behaviours more frequently. Among other things, symptoms of suppression of emotions, somatic complaints and risk behaviour are recorded. The measurement instruments have been validated in various countries and cultures (34, 38–40).

However, some of the depression instruments differ considerably in the symptoms assess (25). For example, while the GMDS asks about hyperactivity, this symptom is missing in the MDS. At the same time, the MDS asks about loss of interest, which the GMDS does not record. In addition to the differences in the composition of the depression instruments, the questionnaires also have methodological weaknesses. For example, the MDS was validated on a small and exclusively male sample (37). However, the MDRS-22 only assesses male-specific symptoms of depression and ignores conventional symptoms (26, 41). The first studies on a newly developed gender-sensitive depression instrument were also recently conducted in Germany. With the Gender-Sensitive Depression Screening (GSDS) (42, 43), a multidimensional depression screening was developed that asks about male-specific and conventional depression symptoms. Symptoms in the areas of stress perception, depressive symptoms, aggressiveness, emotional control, risk behaviour and alcohol consumption are recorded. However, it still appears relatively long with 25 items. A validation of the screening in a mixed-sex sample with explicitly male-specific coping mechanisms, such as alcohol consumption, is also missing (43).

Nevertheless, health research is increasingly calling for gender-sensitive assessments in order to manage preventive and rehabilitative resources more effectively (42, 44–46). Overall, however, there are only a few evidence-based gender-sensitive depression screening instruments; and this gap is also present in Germany (47). At the same time, short forms of survey instruments and screenings are important due to time restrictions and cost-effectiveness of primary care (48). The present study aims to close this research and assessment gap.

### 1.1 Aims of the current study

In line with these aforementioned considerations, the current study aims to validate a German screening instrument for gender-sensitive depression assessment, utilising the German version of the GIDS. Since validation studies to test external validity are ideally conducted on multiple independent samples, this validation is carried out with two separate samples.

In sample 1, we examine the factor structure of the translated GIDS using exploratory factor analysis on a large German-speaking mixed-sex online sample. In this sample, we ask “what is your sex?”. In sample 2, we test the factor structure found using confirmatory factor analysis on a large German-speaking mixed-sex sample with problematic alcohol consumption from a prevention study. In this sample, we ask participants to indicate their sex.

This results in the following research questions which we examine successively in sample 1 and then in turn in sample 2:

1. Which factors that can be clearly distinguished from each other in the GIDS screening version?
2. What are the psychometric characteristics of the screening version?
3. What are the sex differences in the total score of the screening version and at factor level?
4. What is the relationship between depression symptoms and age?

## 2 Methods

### 2.1 Participants and procedure

#### 2.1.1 Sample 1

The sample (N = 1173) was recruited online as part of a thesis (49) via various psychology forums (including Facebook), Germany-wide depression networks, and mailing lists at Philipps University of Marburg were also used. Participation in the online survey was possible between 14.05.2014 and 22.09.2014 without further inclusion or exclusion criteria. This study was conducted in compliance with the Declaration of Helsinki. Participation was based on informed consent. The data protection regulations were observed. At one measurement time point, sociodemographic information, symptoms of depression using the common Beck’s Depression Inventory in revised form (50), somatic complaints using the Brief Symptom Inventory (51), information on inattentive and impulsive behaviours using the Conners’ Adult ADHD Rating Scales (52), symptoms of substance dependence using the Substance Abuse and Mental Illness Symptoms Screener (53), and antisocial behaviour using questions on antisocial personality disorder from the Structured Clinical Interview Questionnaire according to DSM-IV (54) were collected. In addition, male-typical and conventional depression symptoms were assessed in the study using the translated and adapted version of the Gender Inclusive Depression Scale (GIDS) (25). The study was approved by the local ethics committee of the Department of Psychology at Philipps University Marburg under file number 2014-21k.

#### 2.1.2 Sample 2

With the support of the internet-based six-week training course “Cleverly drink less”, participants (N = 418) with problematic alcohol consumption were to be enabled to induce a sustainable change in the behaviour of their consumption, as a result of a systematic monitoring of the substance consumption and the identification of emotions and situations connected to this activity. The prevention study was developed as part of the large-scale EU Innovation Incubator project. To test the effectiveness, a randomized controlled trial with three conditions (intervention 1: minimally guided; intervention 2: unguided; waiting control group) was implemented in a longitudinal format with three measurement points. At measurement time t0, a screening was carried out to check whether interested participants met the inclusion criteria of the study. Current alcohol addiction or another addiction disorder as well as suicidal thoughts were exclusion criteria. This study was conducted in compliance with the study protocol (55), and the Declaration of Helsinki. The data protection regulations were observed. The sample was recruited from June 2014 to February 2016 via the website of the GesundheitsTrainings.Online (GET.ON) project and via the member magazines and websites of BARMER GEK and Kaufmännische Krankenkasse. The study was approved by the local ethics advisory board of the Leuphana University of Lüneburg under the file number Boss201404_OT and registered in the German clinical trials register (No. DRKS00006105). For a detailed description of the training program, the study procedure and the results of the study, please refer to Boß et al. (56). In the present study, only extracts of the data from the screening phase (here sociodemographic information and data from the Alcohol Use Disorders Identification Test (AUDIT) (57)) and of data from the baseline survey before randomisation of the participants to the three groups (Depression Anxiety Stress Scales (DASS-21) (58) and the data from the GIDS) were used. The sample characteristics of the two samples are shown in **Table 1**.

**Table 1:** Characteristics of the two samples in terms of sex, age, educational level and partnership status with percentages and standard deviations in brackets.

	Sample 1 (*N* = 1173)		Sample 2 (*N* = 418)	
	Men	Women	Men	Women
*N*	350 (29.8%)	823 (70.2%)	170 (40.7%)	248 (59.3%)
Age (years)	28.55 (9.7)	27.58 (9.3)	47.27 (11.0)	47.70 (8.7)
Level of education				
Basic Schooling	4 (1.1%)	2 (0.2%)	14 (8.2%)	7 (2.8%)
High school diploma	13 (3.7%)	50 (6.1%)	49 (28.8%)	74 (29.8%)
Vocational baccalaureate	31 (8.9%)	62 (7.5%)	107 (62.9%)	167 (67.3%)
A-levels	169 (48.3%)	414 (50.3%)		
University degree	131 (37.4%)	295 (35.8%)	(no answer option)	(no answer option)
No qualification	2 (0.6%)	0	0	0
Partnership status				
Single	299 (85.4%)	698 (84.8%)	28 (16.5%)	58 (23.4%)
Married/registered partnership	42 (12.0%)	93 (11.3%)	119 (70.0%)	138 (55.7%)
Separated/divorced/widowed	9 (2.6%)	32 (3.9%)	23 (13.5)	52 (20.9%)

### 2.2 Assessments

Gender Inclusive Depression Scale (25).

For the construction of the GIDS please refer to **Appendix 1** or to the study by (25). In the study by (25), the GIDS has an acceptable internal consistency (Cronbach’s alpha) of .78. Reliabilities for the individual constructs are not reported in the study. With regard to the convergent validity of the procedure, a high correlation with depression (*r* = .85) as well as medium correlations with alcohol and substance abuse (*r* = .35 and *r* = .32 respectively) and small correlations with impulse control disorders (*r* = .23) were found. No gender differences were found in the symptom severity of the sample, meaning that the measurement instrument appears to fulfil its research intention.

The version of the GIDS as used herein was translated into German as part of the work by (49). The response format was simplified to the extent that respondents could now only choose between “yes”, “no” and “I don’t know” for the majority of questions. The “refuse to answer” option was removed because we assumed that the participants would readily provide information on the symptom areas they were asked about due to the voluntary nature of their participation in the study. All other response formats were maintained. The time frame to which the enquired behaviours refer was also changed. It was adapted to the DSM-IV criteria and referred to the last two weeks before participation in the study in order to be able to report point estimates rather than lifetime estimates of depression. When evaluating the items, “I don’t know” answers were counted as “no” answers in accordance with the analysis in the original work; this means that only the actual consent with items is counted. One point is awarded for each consent (“true” or “yes”) or each high frequency of occurrence (“often”, “sometimes” or “almost always”, “very often”). For the calculation of the individual total values at factor level, we followed the original concept of the GIDS. Accordingly, each participant can only receive a maximum of one point per factor, even if they agree with more than one item. The total score of the screening version is calculated by adding up the individual factors.

#### 2.2.1 Additional assessments in sample 1

##### 2.2.1.1 Beck’s Depression Inventory in revised form (BDI-II)

With 21 items, the Beck’s Depression Inventory in its revised form (BDI-II) by (50) is a globally established and economical self-assessment procedure for quantitatively recording the severity of depressive symptoms. With an internal consistency (Cronbach’s alpha) of .90 in a non-clinical sample and .91 in an internet sample, the measurement accuracy of the instrument can be described as high.

##### 2.2.1.2 Brief Symptom Inventory (BSI)

The Brief Symptom Inventory by Derogatis (BSI) is used to economically record subjective impairments caused by physical and psychological symptoms and is the short form of the Derogatis symptom checklist (60). In the current study, we only look at the Global Severity Index, which measures basic psychological stress. In a norm sample of healthy adults (*n* = 600), a Cronbach’s alpha of .92 was reported for this index (51).

##### 2.2.1.3 Conners’ Adult ADHD Rating Scales (CAARS)

The Conners’ Adult ADHD Rating Scales (CAARS) by (52) are a German-language adaptation of the English-language Conners’ Adult ADHD Rating Scales by (61). Here, the economic screening version with 30 items was used in its self-report form. The internal consistency in the mixed-sex control group was .94 for all scales in the long version, .90 for the inattention scale and .89 for the hyperactivity scale. There is no internal consistency for the ADHD total scale, which consists of the combined inattention and hyperactivity scales and which we used in our study, as there is for the screeening version.

##### 2.2.1.4 Substance Abuse and Mental Illness Symptoms Screener (SAMISS)

The Substance Abuse and Mental Illness Symptoms Screener (SAMISS) by (53) is an economical screening procedure developed for alcohol and substance abuse as well as general mental disorders, which is used specifically to record these illnesses in HIV-positive people. In the present study, only the questions on substance dependence were adopted from the SAMISS. The SAMISS has been relatively under researched due to its originally limited field of application. (53) validated the questionnaire on the SKID-I (54) and found high values for sensitivity and moderate values for specificity for both areas of the questionnaire. The authors of the study came to the conclusion that positive screenings should be explored in particular detail in a further step due to their limited specificity. To our knowledge, no data are available on the internal consistency of the SAMISS. In the present study, the SAMISS was expanded to include a question from the Wender-Reimherr Interview (WRI), which is part of the Homburg ADHD scales for adults (62). The question “Do you regularly use drugs? If yes, which drugs?” should be answered using a four-level response format from “0 = no” to “1 = slightly” and “2 = clearly” to “3 = cannot be assessed”. The question belongs to the accessory questions and is not included in the evaluation of the WRI, so that no data on internal consistency is available.

##### 2.2.1.5 Structured Clinical Interview Questionnaire according to DSM-IV (SKID-II)

The Structured Clinical Interview for DSM-IV Axis-II (SKID-II) by (54) is a procedure for diagnosing twelve personality disorders listed in the Diagnostic and Statistical Manual of Mental Disorders (DSM-IV; American Psychiatric Association; German: Saß, Wittchen & Zaudig). In this study, only items from the questionnaire for the diagnosis of antisocial personality disorder were used. This personality disorder is characterised by a pattern of disregard and violation of the rights of others. The structured and largely standardised specification of the SKID-II contributes to the objectification of the diagnosis. To our knowledge, there are no studies on the internal consistency of the SKID-II questionnaire.

#### 2.2.2 Additional assessments in sample 2

##### 2.2.2.1 Alcohol Use Disorders Identification Test (AUDIT)

The Alcohol Use Disorders Identification Test (AUDIT) was developed in 1989 on the initiative of the WHO by Babor et al. and is a self-report-based screening procedure that can be used in various countries and cultures to identify people with high alcohol consumption or hazardous drinking habits. In general population a Cronbach’s alpha of .75 (63) shows a moderate internal consistency; in a study of persons with a lifetime or current diagnosis of alcohol misuse and/or dependence, the internal reliability of the AUDIT was .86 (64).

##### 2.2.2.2 Depression Anxiety Stress Scales (DASS-21)

The Depression Anxiety Stress Scales by (58) are an economical self-assessment method for recording symptoms of depression, anxiety and stress in the last week before the survey. The DASS-21 has an internal consistency (Cronbach’s alpha) in non-clinical populations of .88 for the depression scale, .82 for the anxiety scale and .90 for the stress scale (65).

### 2.3 Statistical analyses

All statistical analyses were carried out using the SPSS version 27 program (Statistical Package for Social Sciences) for Windows, which contains Amos, a program for structural equation modelling. The raw data of both samples were subjected to a general data screening. Individuals with missing data and outliers were removed.

#### 2.3.1 Analyses in Sample 1

In Sample 1, the construct validity of the GIDS was analysed in terms of content validity using several exploratory factor analyses with Varimax rotation and successive item selection to ensure integrity of factor structure (66) and to derive the screening version. As we wanted to develop a screening version with high practical relevance, we followed a recommendation by (67) and only included items with a factor loading of >.5 in the screening version. As intercorrelations between the various factors of the GIDS additionally validate the multidimensionality of the measurement instrument, we calculated these. We determined the reliability using Cronbach’s alpha for the total score and for factors with more than two items. The reliability for the factors with two items was calculated using the Spearman-Brown coefficient. To examine criterion validity, we correlated the screening version with the BDI-II to examine convergent validity and with the BSI, the CAARS, the SAMISS and the questions on antisocial personality disorder from the SKID-II questionnaire for the DSM-IV to determine discriminant validity. In order to check whether the latent construct is equally captured by the items used for men and women, we carried out tests for measurement invariance with Amos. For this purpose, we used the confirmatory factor analysis in the multigroup analysis. As there is a violation of the multivariate normal distribution in our data, we used the unweighted least squares (ULS) estimation method to test for measurement invariance. Measurement invariance is a prerequisite for comparing latent means between different groups. As we were interested in the feasibility of mean comparisons between the two sexes for further analysis, we checked whether configural and metric invariance was present. The further necessary check of scalar invariance cannot be tested with the ULS estimation method. We therefore restricted ourselves to configural and metric invariance. Configural measurement invariance refers to an invariant, equivalent factor structure. In the case of metric measurement invariance, the non-standardised loadings of the manifest variables are adjusted across the groups in addition to the configural invariance. (68). For the calculation, we used the step-up approach (69). We started with the least restrictive form of measurement invariance and successively increased the restriction requirements in the models. We compared the changes in the fit measures. Differences in the fit measures should not be greater than.01 (70). Firstly, we calculated the Root Mean Square Residuals (RMR), which measures the mean absolute value of the covariance residuals (71). Values below .05 indicate a good fit (72), while a value of 0 indicates a perfect fit (73); the smaller this value is, the better. The Goodness of Fit Index (GFI) is a measure of the proportion of variance and covariance that a particular model is able to explain. The Adjusted Goodness of Fit Index (AGFI) corrects the GFI by the degrees of freedom of the model. The Parsimony Goodness of Fit Index (PFGI) takes into account both goodness of fit and model parsimony. Values of .50 are frequently observed (74) and this value is typically lower than other indices and sensitive to model size (73). The Normed Fit Index (NFI) compares our proposed model with a model in which there are no interactions between the variables. In the sample, sex effects were calculated using a t-test for independent samples with the screening version of the GIDS and its factors. In addition, the sample was analysed for age effects using correlation calculations. For each analysis, we calculated the effect sizes and evaluated them according to (75, 76).

#### 2.3.2 Analyses in Sample 2

To check whether the data of the participants from Sample 2 led to a comparable model fit to the Sample 1, we calculated a confirmatory factor analysis with Amos. The goodness of fit was calculated. We used unweighted least squares (ULS) for estimation as this makes no assumptions about the distribution (72) and our data showed violations of the normal distribution assumption. The resulting model had 80 degrees of freedom, which is a small number. The more degrees of freedom a model has, the greater the probability that the model will be rejected. If such a model is not rejected, then the values obtained are considered very robust (72). We carried out various analyses. We report the same fit indices as described above. We determined the reliability with Cronbach’s alpha for the total score and those factors with more than two items. The reliability for the factors with two items was calculated using the Spearman-Brown coefficient. To test criterion validity, we correlated the screening version with the DASSS-Depression to determine convergent validity and with the DASS-Stress, the DASS-Anxiety and the AUDIT to calculate discriminant validity. We also checked the measurement invariance in this sample using the same procedure as described for sample 1 above. In the sample, sex effects were calculated using a t-test for independent samples with the screening version of the GIDS and its factors. In addition, the sample was analysed for age effects using correlation calculations. For each analysis, we calculated the effect sizes and evaluated them according to (75, 76).

## 3 Results

### 3.1 Sample 1

#### 3.1.1 Research question 1)

##### 3.1.1.1 Exploratory factor analysis

After checking the prerequisites for conducting exploratory factor analyses, the GIDS was successively subjected to several factor analyses (main axis analyses with varimax rotation), excluding weakly loading items. Initially, a 6-factor solution was obtained according to the eigenvalue criterion. Subsequently, items with factor loadings <.5 were successively removed.

A principal axis factor analysis was conducted on the remaining 15 items with orthogonal rotation (Varimax). The Kaiser-Meyer-Olkin measure verified the sampling adequacy for the analysis, KMO = .882 (‘meritorious` according to 77), and all KMO values for individual items were greater than .555, which is above the acceptable limit of .5 (78). The Bartlett’s test of Sphericity was significant (*p* <.001), indicating that correlations between items were sufficiently large for performing a principal axis factor analysis. An initial analysis was run to obtain eigenvalues for each factor in the data. Five factors had eigenvalues over Kaiser’s criterion of 1 (79, 80) and in combination explained 65.28% of the variance. The scree plot was ambiguous and showed inflexions that would justify retaining either 1 or 5 factors. We retained 5 factors because of the large sample size, the convergence of the scree plot and Kaiser’s criterion on this value and content considerations, whereby factors 2, 3, 4 and 5 each comprised only two items. **Table 2** shows the factor loadings after rotation and the final screening version of the GIDS with 15 items (GIDS-15). The items that cluster on the same factor suggest that Factor 1 represents conventional depression symptoms, Factor 2 represents stress perception, Factor 3 represents anxiety, Factor 4 aggressiveness and Factor 5 substance use. The intercorrelations were ≤.45.

**Table 2:** Summary of exploratory factor analysis results for the GIDS-15-Items (*N* = 1173).

	Rotated Factor Loadings				
GIDS Items	1	2	3	4	5
Factor 1: “Depressive symptoms”					
How often have you felt worthless in the last two weeks?	.73				
I often feel empty inside.	.70				
Have there been several days in the last two weeks when you thought that your life had no meaning and everything was worthless?	.68				
Have there been several days in the last two weeks when you have felt sad, empty or depressed for most of the day?	.68				
Have there been several days in the last two weeks when you’ve spent most of the day discouraged about how things are going to turn out in your life?	.65				
Have there been several days in the last two weeks when you have lost interest in most of the things you used to enjoy (such as work, hobbies, personal relationships)?	.60				
How often have you felt exhausted during the last month for no apparent reason?	.51				
Factor 2: “Stress perception”					
How often did you feel tense during the last two weeks?		.71			
How often have you suffered from mental stress in the last two weeks?		.61			
Factor 3: “Anxiousness”					
Have you worried more in the last two weeks than most people with the same problems?			.73		
Have you been more nervous or anxious in the last two weeks than most people with the same problems?			.64		
Factor 4: “Aggressiveness”					
Have you had a fit of rage in the last two weeks where you suddenly lost control and threatened to hurt or hit someone?				.68	
Have you had a fit of rage in the last two weeks where you suddenly lost control and hit or tried to hit someone?				.52	
Factor 5: “Substance use”					
Have you consumed so much alcohol or other drugs in the last two weeks that your family or friends have worried about you or repeatedly complained?					.63
Have you consumed so much alcohol or other drugs in the last two weeks that it has interfered with your duties at work, school or home?					.54
Eigenvalues	4.98	1.39	1.27	1.08	1.07
% of explained variance	33.23	9.27	8.47	7.18	7.13
*α*/Spearman-Brown coefficient	.87	.70	.72	.53	.51

GIDS, Gender Inclusive Depression Scale. *α* was only calculated for factor 1. Spearman-Brown coefficient was calculated for factors 2, 3, 4 and 5. Only factor loadings above.5 are mapped.

#### 3.1.2 GIDS-15 analyses

##### 3.1.2.1 Research question 2)

In order to determine internal consistency, Cronbach’s alpha was calculated for the GIDS-15 screening version. The total scale resulted in a value of .85 that is described as good. Factor 1 (conventional depressive symptoms) has an equally satisfactory value of .87. Spearman-Brown coefficients were calculated for the other subscales (81). A Spearman-Brown coefficient of .70 was calculated for Factor 2 (stress perception); .72 for Factor 3 (anxiety); .53 for Factor 4 (aggressiveness); and .51 for Factor 5 (substance use). In general, there are no specific guidelines in the literature as to whether this level of value is good or acceptable for factors consisting of only two items (81). Comparisons with other similar scales are recommended and it is pointed out that procedures for screening purposes such as the present GIDS-15 do not come close to the reliability of detailed procedures due to their brevity (82). In this respect, we consider the level of the coefficients for Factor 2 and Factor 3 to be acceptable and for Factors 4 and 5 to be sufficient. The reliabilities are shown in **Table 2**.

Correlations between the GIDS-15 and the external criteria BDI-II, BSI, CAARS, SKID-II and SAMISS were calculated by using Spearman’s Rho non-parametric rank correlation coefficient due to the lack of normal distribution of the data (**Table 3**). With a significant correlation of *r_s_* = .67, *p* <.001, *n* = 1173 between the GIDS-15 and the BDI-II, the convergent construct validity for depression is confirmed. The GIDS-15 also correlates significantly with the BSI, *r_s_* = .71, *p* <.001, *n* = 1173. The correlation between the GIDS-15 and the CAARS is significant, *r_s_* = .46, *p* <.001, *n* = 1173. The GIDS-15 correlates significantly with the sum score of the SAMISS, *r_s_* = .24, *p* <.001, *n* = 1173. A significant result is shown for the correlation between GIDS-15 and for the questions on antisocial behaviour according to the SKID-II questionnaire, *r_s_* = .13, *p* <.001, *n* = 1173.

**Table 3:** Correlations examining the criterion validity of the GIDS-15 screening version in sample 1 using Spearman’s Rho nonparametric rank correlation coefficient.

	1	2	3	4	5	6
1. GIDS-15	1					
2. BDI-II	.67**	1				
3. BSI	.71**	.87**	1			
4. CAARS	.54**	.69**	.73**	1		
5. SKID-II	.13**	.15**	.19**	.27**	1	
6. SAMISS	.24**	.18**	.22**	.29**	.24**	1

GIDS, Gender Inclusive Depression Scale; BDI-II, Beck’s Depression Inventory in revised form; BSI, Brief Symptom Inventory; CAARS, Conners’ Adult ADHD Rating Scales; SKID-II, Structured Clinical Interview according to DSM-IV; SAMISS, Substance Abuse and Mental Illness Symptoms Screener. **significant at *p* <.001. Relevant results are highlighted in bold.

##### 3.1.2.2 Research question 3)

To determine the equivalence between the models between men and women in sample 1, a confirmatory multi-group factor analysis was carried out using the ULS method. Confirmatory invariance was supported, indicating that the model did not generally discriminate between men and women (70). The resulting coefficients were RMR = .006, GFI = .995, AGFI = .992, PGFI = .663, NFI = .992, and RFI = .989. Further tests of measurement invariance revealed equal factor loadings in these two groups, so metric invariance was assumed. The resulting coefficients were RMR = .006, GFI = .994, AGFI = .992, PGFI = .704, NFI = .991, RFI = .989. Factor structure and loadings therefore did not differ between men and women in sample 1.

With regard to the screening version of the GIDS with 15 items, no significant sex differences can be observed in the overall score. Women (*M* = 2.04, *SD* = 1.030, *n* = 823) and men (*M* = 1.95, *SD* = 1.135, *n* = 350) showed comparable values on the overall scale (*t*(1171) = -1.248, *p* = .212). The individual subscales (factors) showed the following sex-specific distributions: For Factor 1, which determines conventional depressive symptoms, there was no significant difference between the sexes (*t*(1171) = -1.241, *p* = .215). However, there was a significant difference for Factor 2, which is intended to measure stress perception (*t*(1171) = -2.170, *p* = .030, *d* = -.145); women reported significantly more stress-related symptoms (*M* = .84, *SD* = .37, *n* = 823) than men (*M* = .79, *SD* = .41, *n* = 350). A significant sex difference was also found for Factor 3, which determines anxiety (*t*(1171) = -2.045, *p* = .041, *d* = -.128); women reported significantly more anxious symptoms (*M* = .34, *SD* = .48, *n* = 823) than men (*M* = .28, *SD* = .45, *n* = 350). There was no significant difference between the sexes for Factor 4, which is intended to measure aggression (*t*(1171) = -.226, *p* = .821). Factor 5, which is intended to measure substance use, showed a significant difference between the sexes (*t*(1171) = 3.432, *p* = .001, *d* = .250); men reported significantly more substance use (*M* = .13, *SD* = .34, *n* = 350) than women (*M* = .06, *SD* = .24, *n* = 823). The results are shown in **Table 4**.

**Table 4:** Descriptive statistics and group comparisons between women and men in mean values in GIDS-15 total and factors in the respective samples.

	Sample 1			Sample 2		
Factor	Men (*n* = 350) *M* (*SD*)	Women (*n* = 823) *M* (*SD*)	Statistics	Men (*n* = 170) *M* (*SD*)	Women (*n* = 248) *M* (*SD*)	Statistics
GIDS-15 total mean value	1.95 (1.14)	2.04 (1.03)	*t*(1171) = -1.248, *p* = .212	1.84 (1.3)	2.18 (1.20)	*t*(416) = -2.716, *p* = .007, *d_Cohen_* = -.273
Factor 1 “Depressive symptoms” mean value	.74 (.45)	.76 (.43)	*t*(1171) = -1.241, *p* = .215	.63 (.48)	.74 (.44)	*t*(416) = -2.333, *p* = .020, *d_Cohen_* = -.240
Factor 2 “Stress perception” mean value	.79 (.41)	.84 (.37)	*t*(1171) = -2.170, *p* = .030, *d_Cohen_* = -.145	.61 (.49)	.80 (.40)	*t*(416) = -4.336, *p* <.001, *d_Cohen_* = -.434
Factor 3 “Anxiousness” mean value	.28 (.45)	.34 (.48)	*t*(1171) = -2.045, *p* = .041, *d_Cohen_* = -.128	.19 (.39)	.24 (.43)	*t*(416) = -1.323, *p* = .187
Factor 4 “Aggressiveness” mean value	.03 (.18)	.03 (.18)	*t*(1171) = -.226, *p* = .821	.04 (.19)	.04 (.19)	*t*(416) = -.054, *p* = .957
Factor 5 “Substance use” mean value	.13 (.34)	.06 (.24)	*t*(1171) = 3.432, *p* = .001, *d_Cohen_* = .250	.38 (.49)	.36 (.48)	*t*(416) = .488, *p* = .626

*GIDS*, Gender Inclusive Depression Scale; *M*, mean; *SD*, standard deviation.

##### 3.1.2.3 Research question 4)

As the normal distribution assumption was violated in our data, we used Spearman’s Rho nonparametric rank correlation coefficient to calculate the correlations between the GIDS-15 score and age. In the overall group, a significant negative correlation was found between the GIDS-15 score and age (*r_s_* = -.10, *p* = .007, *n* = 1173). Separated by sex, there is a significant negative correlation between age in women and the value of the total score (*r_s_* = -.14, *p* <.001, *n* = 823). In men, there is no significant correlation between age and the value in the total score of the screening version GIDS-15 (*r_s_* = -.02, *p* = .769, *n* = 350).

### 3.2 Sample 2

#### 3.2.1 Research question 1)

##### 3.2.1.1 Confirmatory factor analysis

The RMR value in our model was .009. The GFI value of .980 means that the model explains 98% of the variance and reflects a good fit to the model (83). The AGFI shows a good fit with .969. Our study shows a PFGI value of .653, which clearly exceeds the value of .50. The NFI in our model is .959, which means a good fit of the model (84). The Relative Fit Index (RFI) is a derivative of the NFI (74) and reached a value of .946, which indicates a good fit to our model (85). Finally, the PRATIO (Parsimony Ratio) with .762 and the Parsimony Fit Index (PNFI) with .731 represent an acceptable fit. Values greater than .60 are considered satisfactory (72).

#### 3.2.2 GIDS-15 analyses

##### 3.2.2.1 Research question 2)

To determine the internal consistency, Cronbach’s alpha was calculated for the screening version. The internal consistency of the GIDS-15 has a Cronbach’s alpha of .81 and can be described as good. Factor 1 (conventional depressive symptoms) has a Cronbach’s alpha of .81 and can be rated as good. Spearman-Brown coefficients were calculated for the other factors. A Spearman-Brown coefficient of .76 was calculated for Factor 2 (perception of stress); .62 for Factor 3 (anxiety); .51 for Factor 4 (aggressiveness); and .22 for Factor 5 (substance use). Values for Factor 2 and Factor 3 are in the acceptable range, in the sufficient range for Factor 4; and insufficient for Factor 5. Because of content-related considerations, factor 5 is nevertheless retained for the following calculations.

Correlations between the GIDS-15 and the external criteria DASS-Depression, DASS-Stress, DASS-Anxiety and AUDIT were calculated using Spearman’s Rho nonparametric rank correlation coefficient due to the lack of normal distribution of the data (**Table 5**). With a significant correlation of *r_s_* = .56, *p* <.001, *n* = 418 between the GIDS-15 and the DASS-Depression, the convergent criterion validity for depression is confirmed. The GIDS-15 also correlates significantly with DASS stress, *r_s_* = .64, *p* <.001, *n* = 418. The correlation between the GIDS-15 and DASS anxiety is significant, *r_s_* = .44, *p* <.001, *n* = 418. The GIDS-15 correlates significantly with the sum score of the AUDIT, *r_s_* = .41, *p* <.001, *n* = 418.

**Table 5:** Correlations examining the criterion validity of the GIDS-15 screening version in sample 2 using Spearman’s Rho nonparametric rank correlation coefficient.

	1	2	3	4	5
1. GIDS-15	1				
2. DASS_Depression	.56**	1			
3. DASS_Stress	.64**	.62**	1		
4. DASS_Anxiety	.44**	.50**	.58**	1	
5. AUDIT	.41**	.37**	.33**	.33**	1

GIDS, Gender Inclusive Depression Scale; DASS_Depression, Depression Anxiety Stress Scales, subscale Depression; DASS_Stress, Depression Anxiety Stress Scales, subscale Stress; DASS_Anxiety, Depression Anxiety Stress Scales, subscale Anxiety; AUDIT, Alcohol Use Disorders Identification Test. **significant at *p* <.001. Relevant results are highlighted in bold.

##### 3.2.2.2 Research question 3)

In sample 2, we checked the measurement invariance between men and women. Configural invariance was supported so that a comparable data structure can be assumed in both groups. The resulting coefficients were RMR = .011, GFI = .973, AGFI = .961, PGFI = .657, NFI = .947 and RFI = .931. The factor loadings were also the same in both groups, so that metric measurement invariance was assumed. The resulting coefficients were RMR = .011, GFI = .970, AGFI = .958, PGFI = .695, NFI = .940 and RFI = .926. Factor structure and loadings therefore did not differ between men and women in sample 2.

Significant sex differences can be observed with regard to the screening version of the GIDS with 15 items. Women (*M* = 2.18, *SD* = 1.201, *n* = 248) have a significantly higher total score than men (*M* = 1.84, *SD* = 1.303, *n* = 170), (*t*(416) = -2.716, *p* = .007, *d* = -.273). The following sex-specific distributions were found in the individual factors: In Factor 1, which determines conventional depression symptoms, there was a significant difference between the sexes (*t*(416) = -2.333, *p* = .020, *d* = -.240); women (*M* = .74, *SD* = .44, *n* = 248) reported more classic depressive complaints than men (*M* = .63, *SD* = .48, *n* = 170). Factor 2, which measures stress-related symptoms, also shows a significant difference between the sexes (*t*(416) = -4.336, *p* <.001, *d* = -.434); women (*M* = .80, *SD* = .40, *n* = 248) reported significantly more symptoms of perceived stress than men (*M* = .61, *SD* = .49, *n* = 170). For Factor 3, which determines anxiety-related symptoms, there was no significant difference between the sexes (*t*(416) = -1.323, *p* = .187), nor for Factor 4 (aggressiveness; *t*(416) = -.054, *p* = .957) and Factor 5, which records substance-related symptoms (*t*(416) = .488, *p* = .626). The results are shown in **Table 4**.

##### 3.2.2.3 Research question 4)

Because the normal distribution assumption was violated in our data, we used Spearman’s Rho nonparametric rank correlation coefficient to calculate the correlations between the total score in the GIDS-15 and age. In the overall group, there was a significant negative correlation between the GIDS-15 score and age (*r_s_* = -.20, *p* <.001, *n* = 418). Broken down by sex, there is a significant negative correlation between age in women and the value in the total score (*r_s_* = -.20, *p* = .001, *n* = 248). In men, there is a significant negative correlation between age and the value in the total score of the screening version of the GIDS (*r_s_* = -.21, *p* = .006, *n* = 170).

## 4 Discussion

The aim of the present study was to validate a screening version for gender-sensitive depression diagnosis in Germany, using the translated GIDS for the first time in two large German-speaking mixed-sex samples.

### 4.1 Research question 1)

The theoretical design approach of the GIDS from the original study (25) could not be confirmed by factor analysis in our study. In the exploratory investigation of the factor structure of the GIDS-15 in a large online sample in sample 1, five factors were extracted. These are: conventional depression symptoms, stress perception, anxiety, aggressiveness and substance use. Loading of the items on the latent factors was high. The factors themselves were moderately correlated. As expected, it is thus possible to extract clearly definable classic symptoms as well as additional symptoms that are not typical of depression and more attributable to men. Therefore the GIDS-15 seems to be more comprehensive and seems to reflect the heterogeneity of the depression experience (86). At the same time, the factor “conventional depression symptoms” has extraordinary factor-analytical relevance, while the other four factors are relatively equal in terms of their share of explained variance, but are of secondary importance. On the one hand, the factor “conventional depression symptoms” has almost half of all the items ultimately included in the screening version and accounts for around half of the explained variance of the screening version. On the other hand, the factor combines items that depict different constructs (e.g. depressive mood, lack of interest) in the original study (25). Thus, the significance of conventional depression symptoms is stronger in our study than in another study on the construction and validation of a German-language gender-sensitive depression screening (42), but this is might be due to the original construction and conception of the GIDS (25). According to this design, classic and male-typical depression symptoms are included in roughly equal proportions, whereas in the GSDS, prototypical depression symptoms are included proportionally (42). The other four extracted factors (stress perception, anxiety, aggressiveness and substance use) have already been considered in other gender-sensitive depression survey instruments (15, 16, 37), which seems to underline their relevance in the context of gender-sensitive depression diagnostics. However, it is noticeable in our study that the substance use subscale has a subordinate significance as an indicator of depression compared to the other subscales due to lower explained variance. In the GSDS (42, 43), the alcohol consumption subscale is also of secondary importance. Both in our study and in the GSDS studies, the subscales on stress perception and aggressiveness play a more important role according to the proportion of variance explained. In sample 2, the factor structure found in Sample 1 are replicated using a confirmatory factor analysis in a German-speaking mixed-sex sample with problematic alcohol consumption. Overall, the model fit with a latent factor for each of the five factors was very good. The robustness of the results in two different samples thus underlines the external validity of the instrument, especially as the samples differ significantly in some of the characteristics surveyed, such as age or partner status (see **Table 1**). In this respect, this study shows clearly definable factors that include conventional and atypical symptoms of depression. The factors found could serve as an important diagnostic feature for differential diagnosis and thus further empirically support the theoretical construct of male depression. Previously, it has mainly been differential diagnostic limitations that have been discussed (87).

### 4.2 Research question 2)

With 15 items, the GIDS-15 screening version is an economical variant with mostly good to acceptable internal consistencies for the overall scale and the subscales in sample 1. In this sample, Cronbach’s alpha of .85 was comparably high to the original study with .78 in the GIDS version with 25 items (25). In this sample, only the subscales Aggressiveness and Substance Use have just sufficient reliability and are left in the screening version for content considerations. The internal consistencies of the overall scale and the subscales in sample 2 were mostly good to sufficient in view of the number of items per factor, but unacceptable for the substance use subscale. The internal consistency for the substance use subscale was already just sufficient in sample 1. However, the lower internal consistency of the substance use subscale in this sample compared to a general population sample may be due to the heterogeneity of problematic alcohol consumption patterns, leading to greater response variability. This results confirms the fact that the items for this factor should be revised. At the same time, the expectation of group assignment in this sample could have had an influence on reliability (see Limitations). No internal consistencies were specified for the subscales in the original study (25). However, comparable preliminary validations of existing or newly developed gender-sensitive depression instruments in Germany report similarly high or slightly higher reliabilities for their subscales, but with a higher number of items per subscale (34, 42, 43). The low internal consistencies found in this study seem to be due to the small number of items in the subscales. The direction and level of the validation correlations with well-established measurement instruments in sample 1 show results in line with expectations. There is a strong correlation with a well-validated depression instrument, which demonstrates convergent construct validity. The correlation with a psychosomatic complaint instrument is also high, but lower with hyperactive, substance-related and antisocial behaviours, which suggests discriminant construct validity. In sample 2, high correlations with depression and stress scales demonstrate the convergent validity of the GIDS-15 and, in comparison, lower correlations with anxiety and alcohol scales demonstrate the discriminant validity of the procedure. In the original study, however, there was a high correlation with a major depression episode and moderate correlations with alcohol and other drug use as well as with intermittent explosive disorder (25). At the same time, against the background of the correlations in our study, it is unclear whether the GIDS-15 can actually be used to detect depressive symptoms in a gender-sensitive manner or whether the instrument does not rather capture dysfunctional stress management strategies. The differential diagnostic differentiation from other disorders is difficult and has already been discussed in previous studies (87, 88). To clarify this, the investigation should be repeated, taking into account the limitations of this study.

### 4.3 Research question 3)

Measurement invariance was detected in both samples, so that statements can be made about mean value comparisons based on the latent scales between men and women. As expected, no sex differences are found for the total score of the GIDS-15 in sample 1. This result corresponds to the original study (25), although all 25 items were included in the total score there. The result is also consistent with findings from other studies in which traditional depression symptoms and externalising symptoms were combined according to the male gender role (43). At factor level, women report more stress perception compared to men, and men report more substance use compared to women. The results are consistent with the findings from comparable studies (43). The sex distribution of substance use is consistent with the generally higher rates of alcohol and drug use disorders among men (89, 90). The other expected sex differences (women report more depression-typical complaints, men report more depression-atypical, externalising symptoms) are not evident. In sample 2, women have a significantly higher total score than men, which is not in line with our expectations. At the factor level, women report more conventional symptoms of depression as well as stronger perceptions of stress. Beyond that, there are no sex differences, and in particular not for the factors aggressiveness and substance use, which is in line with our expectations. These findings from sample 1 and sample 2 can be interpreted against the background of other studies: On the one hand, previous studies have found a correlation between a basic orientation towards traditional masculinity norms and the report of male-typical complaints and behaviours (35, 91–93). On the other hand, specific characteristics, particularly of Sample 2, can be discussed against this background: Men who approached the study management as part of the prevention study and thus sought help demonstrated behaviour that does not tend to correspond to the traditional male norm orientation (91–94). Corresponding findings indicate that the orientation towards traditional masculinity norms has a negative effect on the utilisation of psychosocial services (29, 95, 96). In contrast, women in this sample are more likely to exhibit male-typical and thus role-incongruent behaviour by reporting problematic alcohol consumption. For example, contrary to the original study (25), in factor aggressiveness women exhibit the same level as men. Previous studies have already published results according to which women also exhibit male-typical behaviour (25, 33, 34, 97). In addition, a recent study has shown that clients with masculine depression have a more critical substance use when compared to clients with non-masculine depression (10). Accordingly, both the results of the overall score and at factor level in our study could have been influenced by attitudes and behaviours that do not conform to gender roles. To test this theory, questions on gender role orientation should be included in future surveys for both women and men, or at least taken into consideration; ideally not only for the area of depressive disorders, but also for other mental disorders, as there are also gender-dependent prevalence distributions for other psychopathological measures, for example for alcohol use disorders (89) or suicides (98). This alone results from the fact that the presented behaviour and the handling of depression or its risk is obviously influenced by the traditional male role orientation (35, 37, 99, 100). Even though we did not use an assessment to measure orientation towards masculinity norms in our study, according to some authors, a symptom pattern consisting of externalising symptoms can be seen as a marker for orientation towards masculinity norms (32, 101). Our study could therefore be a confirmation of the assumed “masculine depression”, which can be understood as a subtype of depression (10). Also, our findings could provide evidence for earlier studies in which it was shown that depression is a heterogeneous disorder (21). At the same time, the results found could also be due to the fact that men show a lower willingness to perceive and subsequently report depressive symptoms than women (102), so that women report more complaints (103). However, there is another far-reaching explanation for the unexpected results regarding sex distribution. This study captures the two binary gender dimensions of female and male. However, it is assumed that there are many more than two gender dimensions (104). The current study focuses primarily on externalising behaviours attributed to biological males. Due to the greater variety of possible gender attributions, there is statistically more variance and consequently less stable gender-specific attributions in our binary screening instrument. As a consequence, sex differences that actually existed in the past may no longer be reflected today when the concept is taken into account. This would mean that research would lag behind reality. Future research should take this into account (see limitations).

### 4.4 Research question 4)

Sample 1 shows a significant negative correlation between the value in the GIDS-15 and age. This age-dependent significant negative correlation is only found in women. This finding can possibly be explained by an exposure to chronic stressors in the university context but also by an increased emancipation of women, who appear more willing to report role-inconsistent behaviour (33, 97), so that younger women report more psychological complaints. It also suggests increasing psychological distress in younger age groups, presumably students in particular, as we recruited sample 1 in a university context, which is similar to previous findings (105). Sample 2 showed a significant negative correlation between the GIDS-15 score and age. In this sample, both sexes showed a significant negative correlation between the GIDS-15 score and age. The findings correspond with earlier studies in which the median age of onset for a major depression episode was in the middle of the 20th year of life across cultures in high-income countries (59). In the work of Martin (86), the application of the GIDS showed that age is a significant predictor of risk cases for depression measured with the GIDS; younger persons are at greater risk of meeting case criteria than older adults (86). Here, too, the increasing willingness to understand and self-identify psychological stress (106) may have had an impact in younger generations.

## 5 Limitations

Despite the important findings reported above, this study has a number of limitations.

First, in both samples, we asked about the gender of the participants in a non-specific manner. We assume that most participants understood this question as a question about their biological sex. However, it is also possible that some participants stated their social gender. In this respect, biological sex and social gender should be recorded separately in future studies, but in principle both should be recorded. Furthermore, we only researched gender in binary terms in our study. We did not take other gender diversities into account, although it is debatable whether purely binary research is still appropriate (104). In this respect, gender beyond the binary should be analysed in further studies. In addition, in our study, no corresponding measurement instrument is used to record gender orientation; in principle, however, this would be desirable (37, 107).

Second, the data collection in sample 1 took place in 2014. The data collection in sample 2 took place until 2016. Despite the age of the data, we consider the study to be important. There is still a research gap in well-validated gender-sensitive depression questionnaires in Germany (43, 47). In addition, the study can contribute to further insights into the concept of male depression.

Third, the online sample is a non-clinical, mixed-sex population sample that was recruited online for reasons of ease of accessibility and potentially greater openness due to anonymity. The prevention sample consists of participants who reported subclinical alcohol consumption and volunteered for the prevention study. Selection and recruitment effects must be discussed in both samples. Sample 1 was primarily recruited in a university context and it cannot be ruled out, for example, that participants suffered from a manifest mental disorder. In sample 2 it should be critically noted that a selection bias may have arisen due to the access routes to the study and the study-related inclusion criteria. Despite these classic, uncontrollable selection effects that limit the generalizability of the results, we consider the sample recruitment for an initial preliminary and exploratory validation study of the GIDS-15 screening version to be justifiable, as we wanted to achieve a large sample size. However, in further studies based on our findings, these aspects should be taken into account and ideally a representative population sample should be used.

Fourth, a further problem is the non-participant bias (108). People who refused to participate or those who opened the survey but did not complete it and were therefore excluded from the data analysis could possibly differ systematically in certain variables from the data in the data set used. Even if participation was voluntary, this selection effect could be significant here.

Fifth, the special nature of sample 2 must also be taken into account for the subscale substance use: it cannot be ruled out that the participants in the prevention study followed the need for social conformity and answered questions about substance use accordingly, possibly in the expectation that certain response behaviour would have an influence on subsequent allocation to the intervention or waiting group (55). This could have had an influence on the reliability.

Sixth, all of the survey methods used were self-assessment methods, which means that despite anonymity, distortions may have occurred due to social desirability, deliberate distortion on the part of the participants or a tendency towards acquiescence. The fact that the external criterion for determining construct validity was also a self-assessment procedure is methodologically critical. We were unable to implement the gold standard of a clinical interview to clarify mental disorders due to the online survey. This point should also be urgently considered in further studies.

Seventh, and finally, the translated GIDS was adapted in the present study in accordance with the criticisms of Martin et al. (25) themselves and Kuehner (87), as well as in the response format and the time frame of reference. As no back-translation was carried out, it is possible that the meaning of the constructs to be surveyed changed as a result of the modifications. The sometimes unacceptable reliability of individual subscales could also be due to the formal changes. Nevertheless, it should be noted that in the original study (25) no reliabilities are reported for the individual factors and Cronbach’s alpha for GIDS-15 was higher in both samples of our study than for GIDS with 25 items in the original study, which can, however, also be attributed to the heterogeneity of the constructs to be recorded.

Overall, some of the limitations described above could be addressed by using more suitable samples.

## 6 Conclusion

In summary, the German-language GIDS-15 proves to be an economical and solid screening instrument for gender-sensitive depression diagnosis despite the limitations mentioned above. In a non-clinical German-speaking mixed-sex sample with conventional depression symptoms, stress perception, anxiety, aggressiveness and substance use, we found five factors that could be clearly delineated by factor analysis and confirmed in a German-speaking mixed-sex subclinical alcohol prevention sample. The reliabilities were largely acceptable. The sex distribution at the level of the total score and the individual factors provided the majority of the expected results. Nevertheless, studies are needed to further validate the GIDS-15, for example whether the instrument is also valid for change in the application of psychological interventions. The questionnaire should also be used in more suitable samples and validated with a strong external criterion.

The study provides important implications for practice and future research. Our study confirms earlier evidence that gender-specific coping patterns should be taken into account in the diagnosis of depression, in addition to the classic symptoms of depression. This also emphasizes the relevance in primary care and of corresponding questionnaires. Further research should therefore focus on the increasing consideration of the gender perspective beyond binary classifications in depression research. The long-term goal should be to measure more inclusive instruments that integrate more than two gender options.

## Data Availability

The datasets presented in this article are not readily available. Requests to access anonymized datasets should be directed to the corresponding author.

## Author’s note

The original study (56), from which we used the data from sample 2 in this further analysis, was funded by the European Union (project number: ERDF: CCI 2007DE161PR001). The funders had no influence on the study design, data collection, analysis, interpretation of the results or the decision to publish the study results.

## Appendix 1

About the GIDS:

The GIDS was created in a two-stage process. First, an extensive literature search was conducted to create a list of alternative symptoms of depression for men. In the second step, this list was then compared with the National Comorbidity Survey Replication questionnaire and its data set (59) to determine whether appropriate variables were available to capture the desired symptoms. Symptoms were not included in the scale for three possible reasons: Symptoms were deemed inappropriate, no equivalent items were available in the data, or the number of individuals for whom data were available for a symptom was too small to be useful for analysis; the authors set the cut-off for the minimum number here at 250 individuals. Accordingly, the GIDS contains a total of 15 symptoms (the number of items per construct is given in brackets): depressive mood (three items), listlessness (two items), fatigue (two items), indecisiveness (one item), restlessness (two items), worthlessness (one item), social withdrawal (one item), irritability (two items), anger attacks/aggressiveness (three items), sleep disturbance (one item), alcohol or substance abuse (three items), risk-taking (one item), hyperacvity (one item), stress (two items) and lack of interest (one item). The item on lack of interest is also part of the "depressive mood" scale. Nineteen items come from the screening part of the NCS-R, three items were taken from the 30-day symptom assessment part and one item each comes from the areas of neurasthenia, personality and mania. Depending on the question, the total of 25 items on this scale are processed on a two-, four- or five-point response format (two-point response format: "true", "false"; four-point response format: "yes", "no", "I don't know", "refuse to answer" or "often", "sometimes", "rarely", "never"; five-point response format: "almost always", "very often", "not very often", "never", "I don't know") according to agreement/disagreement or frequency of occurrence. In the original version of the GIDS, the information relates to the entire lifespan, as the majority of the items originate from the NCS-R study (59).
